# Toward digitally supported self-assessment of patients with idiopathic inflammatory myopathies

**DOI:** 10.1186/s13075-025-03504-z

**Published:** 2025-02-22

**Authors:** Felix Kurt Seese, Pia Roscher, Birte Coppers, Julia Greenfield, Manuel Grahammer, Sebastian Kuhn, Latika Gupta, Georg Schett, Johannes Knitza, Anna-Maria Liphardt

**Affiliations:** 1 Department of Internal Medicine 3, Rheumatology and Immunology, Friedrich-Alexander-Universität Erlangen-Nürnberg and Universitätsklinikum Erlangen, Ulmenweg 18, 91054 Erlangen, Germany; 2 Deutsches Zentrum Immuntherapie, Friedrich-Alexander-Universität Erlangen-Nürnberg and Universitätsklinikum Erlangen, Erlangen, Germany; 3 Institute for Digital Medicine, University Hospital Giessen-Marburg, Philipps University, Baldingerstrasse 1, 35043 Marburg, Germany; 4 ABATON GmbH, Berlin, Germany; 5 Department of Rheumatology, Royal Wolverhampton Hospitals NHS Trust, Wolverhampton, UK

**Keywords:** Idiopathic inflammatory myopathies (IIM), Muscle function assessment, Inertial sensor-based gait analysis, Digital health, Remote monitoring

## Abstract

**Background:**

Manual muscle testing (MMT8), the current gold standard for assessing muscle function in patients with idiopathic inflammatory myopathies (IIM), has notable limitations. This study had three aims (1) to compare MMT8 with inertial sensor-based gait analysis, (2) to evaluate patient-performed functional tests guided by shared decision-making (SDM), and (3) to investigate adherence to electronic patient-reported outcomes (ePROs).

**Methods:**

Gold standard muscle function assessment (MMT8) was performed at baseline (T0) and three months (T1). Additionally, inertial-sensor-based gait analysis was completed at T0 and two standardized upper extremity (Modified Barré test; 10-time arm lift test) and two lower extremity muscle endurance tests (60-second Sit-to-Stand (STS) test; Mingazzini test) were presented to patients to choose from. Through shared decision-making, each patient selected one test for lower and upper extremities and opted to record weekly results on paper or through a medical app. Correlations between gait parameters, functional tests, and MMT8 were analyzed, while agreement between patient- and healthcare professional (HCP)-recorded results at T0 and T1 was assessed. Responsiveness to change was also evaluated.

**Results:**

A total of 28 IIM patients (67.9% female; mean age 57.4 ± 12.9 years) were enrolled. Moderate correlations were observed between gait parameters and MMT8, such as walking speed (*r* = 0.545, *p* = 0.004) and stride length (*r* = 0.580, *p* = 0.002). All patients selected the Modified Barré test for assessing upper extremity function and 60.7% of patients chose the Mingazzini test for lower extremity function. Agreement between patient- and HCP-recorded functional test results was excellent at baseline and after three months (ICC 0.99–1.00). Functional tests demonstrated strong correlations with MMT8, particularly for the Mingazzini test (*r* = 0.762, *p* = 0.002). Patients preferred app-based recording (82.1%) over paper-based methods and weekly ePROs were completed on average 6.9 out of 12 weeks (57.5%).

**Conclusion:**

Patient-performed functional tests are reliable, scalable alternatives to MMT8, with gait analysis providing complementary insights. Digitally supported self-assessments can enhance clinical workflows, remote monitoring, and treat-to-target strategies, empowering patients and improving disease management.

**Supplementary information:**

The online version contains supplementary material available at 10.1186/s13075-025-03504-z.

## Introduction

Idiopathic inflammatory myopathies (IIM) are a group of rare autoimmune diseases marked by progressive, symmetric weakness in the proximal muscles [1–3]. Regular and standardized assessment of muscle weakness is essential to monitor disease activity and progression, evaluate treatment effectiveness, tailor rehabilitation and immunosuppressive therapies [3–6]. Traditionally, these assessments are performed by healthcare professionals (HCP), often requiring patients to travel long distances which is a considerable burden for individuals with muscle function impairments. Manual muscle testing (MMT8) has become the gold standard for assessing muscle function as it is a core component of the International Myositis Assessment and Clinical Studies Group (IMACS) Disease Activity Core Set Measures [7]. A HCP tests the isometric muscle strength of eight muscle groups of the dominant side [5]. However, MMT8 has faced criticism for being time-consuming, insufficiently responsive, subjective, limited to short-term maximum strength measurement, and prone to inter-rater variability [3, 4, 8–10].

The rise of digital health solutions offers new possibilities for patient empowerment, increased objectivity, and continuity in muscle function assessment [11–13]. Data granularity can vary greatly depending on equipment costs and the level of patient burden associated with data recording. Enhanced data availability could support timely treatment adjustments and reduce or increase the efficiency of in-person visits. Improved responsiveness of outcome measures may also facilitate the detection of subtle changes, making it easier to achieve primary outcomes in clinical trials. Ideally, muscle weakness could be measured objectively, passively, and continuously with high granularity by patients at home. While consumer-based activity trackers have shown moderate correlations with disease activity, they often lack the accuracy of detailed gait analysis and are not specifically designed for this purpose [14]. Sensor systems using inertial measurement units (IMUs) offer a promising alternative to traditional, costly gait analysis methods, providing a balance between affordability, data precision, and the ability to collect data in real-world settings. A more scalable and cost-effective approach would allow patients to independently assess muscle function anytime, anywhere, with no additional equipment [8, 10]. Previous studies have demonstrated the feasibility of functional tests as alternatives to the MMT8 [8, 10, 15], however HCP were still required to assess the outcomes. Additionally, these studies lacked a shared decision-making (SDM) component, which could help tailor functional tests to individual needs, foster adherence and offer more personalized and relevant monitoring for IIM patients. Finally, incorporating subjective patient-reported outcomes is essential for achieving a holistic clinical perspective [6]. Measures such as the patient global assessment, which is also included in the IMACS Disease Activity Core Set, provide valuable insights into the patient’s own experience of their disease activity.

This study aimed (1) to compare MMT8 with inertial sensor-based gait analysis, (2) to evaluate patient-performed functional tests guided by shared decision-making (SDM), and (3) to investigate adherence to electronic patient-reported outcomes (ePROs).

## Methods

### Study design and study population

This study was approved by the Institutional Review Board of the Medical Faculty of the University of Erlangen-Nürnberg, Germany (Reg No. 334_18B and 166_18B). All procedures adhered to relevant guidelines and regulations, including the Declaration of Helsinki, and all participants provided written informed consent prior to enrollment. Eligible participants were adults aged 18 years or older, with the ability to walk independently either 4 × 10 m or continuously for 2 min, and meeting the 2017 ACR/EULAR classification criteria for idiopathic inflammatory myopathies (IIM) [16]. The exact diagnoses were obtained from the rheumatologists’ clinical letters. Recruitment occurred from October 2021 to November 2022 at the rheumatology department’s outpatient clinics and ward at University Hospital Erlangen. This longitudinal study spanned over three months, with assessments conducted at baseline (T0) and three months (T1) during routine clinical visits. At each visit, the following IMACS core set measures were recorded: Unilateral MMT8 assessment of the dominant side (total score range: 0–80), laboratory enzymes (Creatine kinase, CK; Aspartate transaminase level, AST; Alanine transaminase level, ALT), Health assessment questionnaire (HAQ) and Patient global activity (PGA). At clinic visits participants underwent a gait analysis using the RehaGait^®^ inertial sensor system (Hasomed GmbH, Magdeburg, Germany) and completed the following questionnaires: Short Form (SF-36) Health Survey, Physical Activity-related Health Competence (PAHCO), Tampa Scale of Kinesophobia (TSK), and International Physical Activity Questionnaire (IPAQ), and the question “Are you deliberately physically active? (walking, Nordic walking, running groups, swimming, cycling, etc.)”. Functional tests (details see below) were selected at T0 with agreement between healthcare provider and patient-recorded results assessed at both T0 and T1. Patients were instructed to record their weekly at-home functional test results on paper or via a medical app. Participants who chose to use the medical app additionally provided a set of electronic patient-reported outcomes.

### Gait analysis

**Fig. 1:**
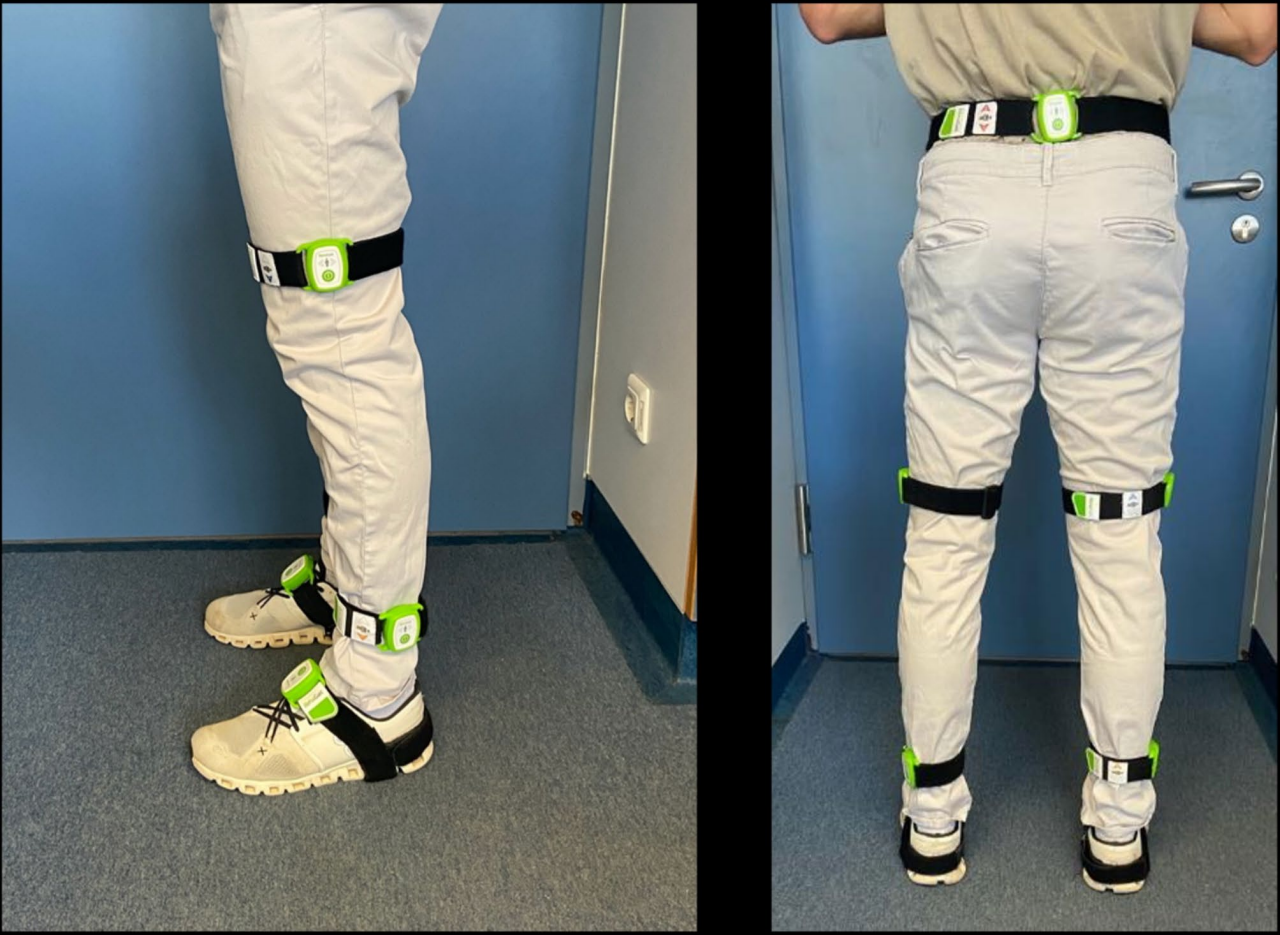
The inertial measurement units (IMUs) of the RehaGait^®^ system

Gait analysis was conducted through a 2-minute walk test. The RehaGait^®^ inertial sensor system was utilized, comprising of seven IMUs, affixed with elastic Velcro bands (Fig. 1). RehaGait^®^ has been previously validated and utilized in multiple studies involving patients with stroke, Parkinson’s disease, and osteoarthritis [17–19]. Sensors were placed on the dorsum of each foot, the lateral lower third of each calf and thigh, and the pelvis at the lumbosacral transition. The system was calibrated before each session following the manufacturer’s instructions.

Key spatiotemporal gait parameters, including distance walked (m), walking speed (m/s²), cadence (steps/min), and stride length (m), were analyzed for the 2-minute walk using the manufacturer’s algorithm. Sagittal kinematics were calculated based on the methodology of Seel et al. [20], encompassing hip range of motion (ROM) during the stance phase, hip angle from initial contact to toe-off (at approximately first 60% of the gait cycle), and knee ROM during the terminal stance phase (at 30–50% of the gait cycle). With provisions to identify and clean outliers caused by sensor malfunctions kinematic data for individual steps were visualized using a custom Matlab (MathWorks Inc., Natick, MA, USA) algorithm adapted from Ismailidis et al. [18]. Single steps were defined as outliers and excluded from analysis if the calculated angle was inverted, showed values outside the anatomical range of the corresponding joint, or the entire signal was shifted after a certain point.

### Functional tests for self-assessment

At T0 participants were presented with four tests to assess upper-extremity (Modified barré test; 10-time arm lift test) and lower extremity muscle endurance (60 s Sit-to-stand (STS); Mingazzini test) from which to choose. The modified version of the original Barré test, used to detect hemiparesis through pronator drift, assesses arm weakness by having participants hold a 1 L (L) (~ 1 kg) bottle at 90° shoulder flexion with their dominant arm [21]. The time until the arm drops, or an evasive movement occurs was recorded (max. 60 s). The 10-times arm lift test requires participants to perform 10 repetitions of raising both arms overhead until the palms touch, then completely lowering them back down [10]. The time to complete the repetitions is measured (max. 45 s). The sit to stand test (STS) instructs participants to stand up from a chair and sit back down as many times as possible, without using their arms for support, recording the number of repetitions performed in one minute (max. 40 repetitions) [8]. The Mingazzini Test involves participants lying in a supine position, flexing their hip and knee joints to a 90° angle, and attempting to maintain this posture as long as possible, with the duration of correct posture recorded (max. 100 s) [8]. Through a shared decision-making process, participants and HCPs collaboratively selected one functional test for both the upper and lower extremities and decided to record the results of the weekly at-home performed functional tests either on paper or via the ABATON medical app (ABATON GmbH, Berlin, Germany). Agreement between healthcare provider and patient-recorded functional test results was assessed at both T0 and T1.

### Electronic patient-reported outcomes

**Fig. 2:**
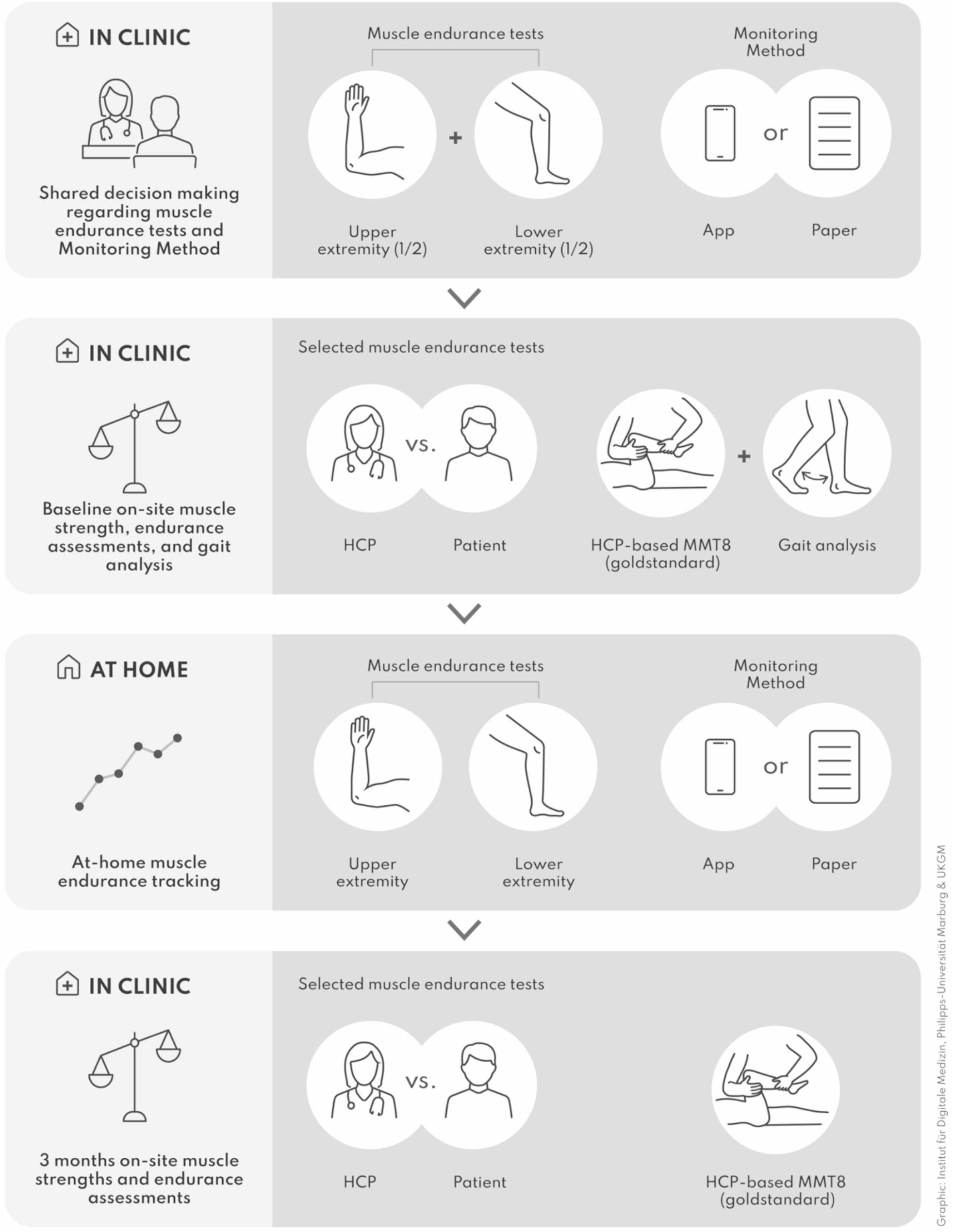
Study flow

Participants who opted to use the medical app reported self-assessed outcomes of the two selected functional tests and reported an additional set of electronic patient-reported outcomes (ePROs), including IMACS patient global disease activity [6], and 7 non-validated questions, including muscle/joint/skin disease activity, overall pain, muscle strength, muscle endurance, and fatigue over the past 7 days, all rated on 0–10 numeric scales (example screenshot of the ABATON app are provided in Supplementary Figure S1). Adherence to ePRO completion was monitored over the 12-week period. A complete overview of the study design is given in Fig. 2.

### Data management and statistical analysis

Research electronic data capture (REDCap, version 14.0.27) was used for data management.

Correlation analyses between gait parameters, functional tests and the MMT8 were performed using the Spearman’s Rank Correlation Coefficient (rho), while correlations between HCP and patients for all functional self-tests were conducted using Pearson’s Product Moment (r). Correlations of 0.1–0.39 are considered weak, 0.4–0.69 are considered moderate, and correlations > 0.7 are considered strong. Statistical significance was set at *p* < 0.05 a priori. All correlations are reported to three decimal places with lower and upper 95% confidence intervals (CI).

Agreement between HCP and patient functional test results was assessed using interclass correlation coefficients (ICC) based on a mean-rating (k = 2), absolute-agreement, 2-way random-effects model. These are reported with confidence intervals as ICC (3,1) [lower CI, upper CI]. The follow-up responsiveness was evaluated using the standardized response mean (SRM) and the standardized mean difference (SMD). The SRM was calculated by dividing the mean change in scores by the standard deviation of that change, the effect size of the SRM was taken as the correlation coefficient between baseline and follow-up measures. SRM effect and SMD values were interpreted as follows: less than 0.5 indicated a mild response, 0.5–0.8 indicated a moderate response, and greater than 0.8 indicated a strong response [8]. The statistical significance of the SRM and SMD were assessed by conducting a Wilcoxon’s Signed Rank test. All statistical tests were performed using R (version 4.4.0; R Core Team 2024) and RStudio (version 2024.02.4).

## Results

### Participants

Twenty-eight patients (19/28 (67.9%) female; mean age 57.4 ± 12.9 years) were enrolled in the study, with the majority of patients being diagnosed with antisynthetase syndrome (Table 1). In total, 26/28 patients completed the three-months evaluation. Two participants were lost to follow-up due to relocation and to high disease activity at follow-up, respectively. Mean (SD) baseline MMT8 score was 69.8 (8.5), and 13/28 (46%) patients presented elevated CK levels.

**Table 1:** Baseline demographics of study population (*n* = 28)

**Demographics**			
	Female, *n* (%)	19 (67.9)	
	Age, years, mean (SD)	57.4 (12.9)	
	BMI, kg/m2, mean (SD)	28.4 (7.4)	
	Alcohol, *n* (%)	15 (53.6)	
	Smoking (currently), *n* (%)	11 (39.3)	
	Prednisone, *n* (%)	11 (39.3)	
	Physiotherapy, *n* (%)	7 (25)	
	Disease duration (since diagnosis), years, median (IQR)	5.5 (11.3)	
**Laboratory results**			
	CK [U/l], mean (SD)	252.9 (292.7)	
	AST [U/l], mean (SD)	35.3 (21.3)	
	ALT [U/l], mean (SD)	40.2 (54.4)	
**Physicial activity**,** health- and disease-related questionnaires**			
	HAQ, 0–3 units, median (IQR)	0.875 (1.3)	
	SF-36, physical functioning, median (IQR)	65 (60)	
	Global disease activity score (0–10 NRS), median (IQR)	40 (58.5)	
	PHACO endurance, median (IQR)	7 (13)	
	PHACO strength, median (IQR)	10 (8)	
	TSK, median (IQR)	38 (9.5)	
	Physically active, *n* (%)	20 (71.4)	
	IPAQ total physical activity MET-min./week, median (IQR)	2544 (2778)	
**Muscle function**			
	MMT8 (max.80), mean (SD)	69.8 (8.5)	
	MMT8 neck flexors, mean (SD)	9.25 (1.0)	
	MMT8 deltoid, mean (SD)	8.75 (1.3)	
	MMT8 biceps, mean (SD)	9.0 (1.0)	
	MMT8 gluteus maximus, mean (SD)	8.3 (1.5)	
	MMT8 gluteus medius, mean (SD)	7.7 (1.5)	
	MMT8 quadriceps, mean (SD)	8.8 (1.5)	
	MMT8 wrist extensors, mean (SD)	9.0 (1.0)	
	MMT8 ankle dorsiflexors, mean (SD)	9.1 (1.0)	
	Modified Barré test [sec.], mean (SD)	51.1 (15.2)	
	STS test [repetitions], mean (SD)	19.7 (6.4)	
	Mingazzini test [sec.], mean (SD)	44.6 (43.8)	
**Diagnoses**			
	Dermatomyositis, *n* (%)	8 (28.6)	
	Antisynthetase syndrome, *n* (%)	9 (32.1)	
	Overlap myositis, *n* (%)	4 (14.3)	
	Polymyositis, *n* (%)	3 (10.7)	
	Interstitial myositis, *n* (%)	2 (7.1)	
	Necrotizing myopathy, *n* (%)	2 (7.1)	
**Spatiotemporal Gait Parameters**			
	Walking speed [m/s2], mean (SD)	1.2 (0.2)	
	Walking cadence [steps/min], mean (SD)	107.7 (11.2)	
	Stride length [m], mean (SD)	1.3 (0.1)	

Abbreviations: IIM, Idiopathic inflammatory myopathy; NRS: Numeric rating scale 0–10; MMT8, manual-muscle-testing; PROs, patient-reported outcomes; HAQ, Health assessment questionnaire; SF-36, Short form-36 health survey, dimension of physical functioning; PHACO, Physical Activity-related Health Competence; TSK, Tampa Scale of Kinesophobia; IPAQ, International Physical Activity Questionnaire; MET-min./week, metabolic equivalent– minutes/week; CK, creatine kinase level (normal range 0-150 U/l); AST, aspartate transaminase level (normal range 10–50 U/l); ALT, alanine transaminase level (normal range 10–50 U/l); IQR, Interquartile range; SD, standard deviation; CI, confidence interval.

### Gait analysis

A moderate positive correlation with MMT8 was observed for both walking speed (*r* = 0.545, *p* = 0.004) and stride length (*r* = 0.580, *p* = 0.002); however, all other gait parameters showed negligible or weak correlations with MMT8 (Table 2).

**Table 2:** Correlations between manual muscle testing results, with gait parameters and functional tests conducted at the baseline visit. Significant correlations are marked in bold

	Correlation coefficient [95% CI]	*p*-value
**Gait parameters**		
Walking speed (meters/seconds)	**0.545** [0.20; 0.77]	0.004
Walking cadence (steps/minute)	0.143 [-0.26; 0.50]	0.485
Stride length (meters)	**0.580** [0.25; 0.79]	0.002
Left knee ROM at terminal stance (°)	-0.072 [-0.45; 0.33]	0.732
Right knee ROM at terminal stance (°)	0.320 [-0.08; 0.63]	0.111
Left hip ROM in stance phase (°)	0.102 [-0.30; 0.47]	0.619
Right hip ROM in the stance phase (°)	-0.205 [-0.55; 0.20]	0.315
**Functional tests**		
Mingazzini (seconds)	**0.762** [0.39; 0.92]	0.002
60 s sit to stand (repetitions)	**0.638** [0.19; 0.87]	0.011
Modified barré (seconds)	**0.456** [0.09; 0.71]	0.017

ROM = range of motion

### Functional self-assessment tests

**Fig. 3:**
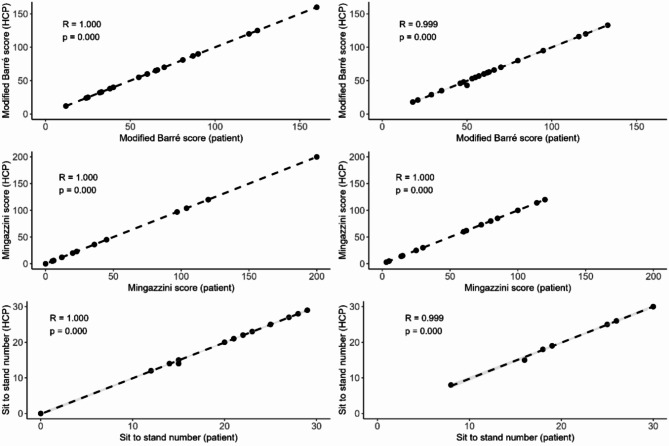
Correlation plots between patients and healthcare professionals for the three functional tests performed at baseline (left) and at follow up (right)

All patients (28/28; 100%) selected the Modified Barré test to evaluate upper extremity muscle function. 17/28 (60.7%) chose the Mingazzini test and 11/28 (39.3%) the 60-seconds Sit-to-Stand test to evaluate lower extremity muscle function. Based on this result, we hypothesized that weaker patients (represented through a lower MMT8 score) chose the Mingazzini test, while stronger patients chose the sit-to-stand test. Differences in MMT8 scores between patients, who chose the Mingazzini test (70.29 ± 8.42) and patients, who chose the sit-to-stand test (70.13 ± 7.14), were evaluated using a Welch‘s t-test. No statistically significant difference in MMT8 score at baseline was found between the two groups (t (25.6) = 0.05, *p* = 0.96). Patient and healthcare professional assessment of in-clinic performed functional tests at baseline and the three month follow-up (Fig. 2) correlated well, ranging from 0.99 to 1.00 (Table 3; Fig. 3). Strong, positive, correlations were observed between the MMT8 and lower-limb functional tests (Mingazzini score: *r* = 0.762, *p* = 0.002; sit-to-stand: *r* = 0.638, *p* = 0.011). The modified Barré score presented a moderate, positive correlation with the MMT8 score (*r* = 0.456, *p* = 0.017; Table 2). Graphical representations of the individual correlations are available in Supplementary Figure S2.

**Table 3:** Correlation coefficients and intraclass correlations coefficients between healthcare professional and patient scores for functional tests. Significant correlations are marked in bold

Assessment time	Functional test	Correlation coefficient [lower CI; upper CI]	*p*-value	ICC [lower CI ; upper CI]
T0	Modified Barré	**1.000** [1.000; 1.000]	< 0.0001	1 [1;1]
T0	Mingazzini	**1.000** [1.000; 1.000]	< 0.0001	1 [1;1]
T0	Sit to stand	**1.000** [1.000; 1.000]	< 0.0001	1 [0.998;1]
T1	Modified Barré	**0.999** [0.999; 1.000]	< 0.0001	0.998 [0.997; 0.999]
T1	Mingazzini	**1.000** [1.000; 1.000]	< 0.0001	1 [1;1]
T1	Sit to stand	**0.999** [0.999; 1.000]	< 0.0001	0.999 [0.997;0.999]

T0 = Baseline; T1 = 3 months follow-up

### Responsiveness to change analyses

The responsiveness to change analysis is presented in Table 4. The direction of change in score varied among the tests. The Modified Barré, Mingazzini and MMT8 scores all showed a mild decrease at follow-up, while the sit to stand score showed a mild increase. Responsiveness to change had the strongest effect for the Mingazzini score, despite this score presenting the lowest SRM (-0.15). The highest SRM was observed in the sit to stand (0.53) with moderate effect. None of the observed changes were statistically significant.

**Table 4:** Analysis of change magnitude at the follow-up assessment compared to baseline of the respective muscle function assessments

Muscle function assessment	*n*	Average change	SMD	SMD effect	SRM	SRM effect	*p*-value
Modified Barré score	26	-7.73	-0.22	Mild	-0.22	Mild	0.338
Mingazzini score	12	-5.58	-0.09	Mild	-0.15	Strong	0.965
Sit to stand	10	2.9	0.42	Mild	0.53	Moderate	0.199
MMT8	26	-2.27	-0.26	Mild	-0.3	Moderate	0.153

SMD = standard mean difference; SRM = standardized response mean

### Monitoring preference and adherence to ePROs

Most patients (23/28; 82.1%) preferred using the medical app over paper to record the functional test results. On average, patients completed weekly ePROs sets (10 questions in total) for 6.9 out of 12 weeks (57.5%).

## Discussion

Manual muscle testing has faced significant criticism, and there has been growing advocacy for incorporating complementary measures, such as functional tests supported by objective sensor technologies, to enhance the value of functional assessments in patients with idiopathic inflammatory myopathies. This study aimed to assess IMU-based gait analysis and patient-performed functional tests as viable methods to assess muscle function in patients with idiopathic inflammatory myopathies. Functional tests demonstrated strong correlations with MMT8 scores and showed equal, if not higher, responsiveness to change compared to the MMT8, indicating their potential as valid muscle function assessment tools. Notably, there was excellent agreement between patient- and HCP-recorded functional test results, demonstrating the reliability of self-assessment. To our knowledge, this is the first study to examine myositis patients’ preferences regarding functional tests. Interestingly, all participants opted for the Modified Barré test to evaluate upper extremity muscle function. Future qualitative studies could investigate the underlying reasons for the preference of certain functional tests over others.

Moderate, positive correlations between functional tests and MMT8 have been previously observed [8]. Our results support previous studies that found correlations between muscle strength, walking speed and stride length in IIM [22, 23]. Furthermore, recent advances in digital medicine have identified 95th percentile stride velocity as an approved digital biomarker in Duchenne muscular dystrophy, highlighting the relevance of gait as a valuable outcome parameter [24]. However, in contrast to the extensive research on gait analysis in conditions such as stroke or Parkinson’s disease, studies specifically addressing gait impairments in myositis patients are limited. The RehaGait^®^ system has not been previously used in an IIM population. This highlights the relevance of our study, which contributes to novel insights by systematically evaluating gait patterns in this patient population. The system correctly detected gait cycles in these patients and quantification of gait parameters was possible in our cohort. Assessing gait is promising, as no extra work or instruction is necessary, as patients naturally walk each day. Given that affordable smartphones, fitness trackers, and smartwatches can provide clinically relevant gait data such as walking speed, acceleration, and stride length [14, 25], the added clinical value of costly and complex gait analysis systems needs further investigation. Notably, the 2-minute walking distance has been shown to be a reliable indicator of muscle strength and can be easily self-administered by patients without the need for expensive equipment [9].

Our results confirm results from Landon-Cardinal et al. that demonstrated a similar responsiveness to change in functional tests compared to that of MMT8 [8]. Unlike their study, our findings indicate that the sit-to-stand test demonstrated the highest responsiveness to change. None of the observed changes reached statistical significance, likely due to the small sample size and the absence of planned treatment interventions to drive measurable improvements. Importantly, our study demonstrated high agreement between patient- and HCP-recorded functional test results, indicating the reliability and accuracy of these patient-performed assessments. This suggests that simple, scalable functional tests could enable patient self-assessment of muscle function, allowing patients to record data at home and share it with their healthcare providers. This approach can save time, enhance the efficiency of in-person visits by providing more informed clinical insights, reduce unnecessary in-person appointments, and facilitate early detection of disease progression. The simplicity of functional tests, which require no costly equipment for test performance or results recording, presents a distinct advantage over more complex tests, such as the functional index [15], which necessitates special wrist weights and precise movement pacing. These requirements restrict its practical use and ease of access for patients. Shared decision-making, enables patients to select preferred functional tests and teaching them proper execution, appears to be a good investment that may increase patient ownership and adherence.

In our study, patient-collected data were not used for clinical decision-making or discussed with patients, which may have impacted adherence. We propose that incorporating the Borg scale to assess perceived exertion, similar to the functional index [15], could enhance the depth of information gathered, and future studies could explore this.

Interestingly, most patients favored using the medical app over paper-based recordings, supporting telemedicine implementation and facilitating easy data export for research [26]. The absence of comprehensive data on disease characteristics and detailed medication exposure represents a limitation of this study. Furthermore the small sample size limits the generalizability of these findings, emphasizing the need for larger validation studies to confirm these results.

## Conclusion

This study demonstrates the reliability of patient-performed functional tests for muscle function assessment in idiopathic inflammatory myopathies. Strong correlations with MMT8 scores and high responsiveness to change confirm their clinical utility. Patients’ preference for app-based recording supports telemedicine solutions for streamlined remote monitoring, while shared decision-making in functional test selection promotes engagement and adherence. Although promising, these findings require validation in larger studies to establish their broader applicability and potential to enhance patient self-management and clinical efficiency.

## Electronic supplementary material

Below is the link to the electronic supplementary material.

Supplementary Material 1

## Data Availability

Data are available from the corresponding author on reasonable request.

## Abbreviations

ACR: Arthritis Care & Research
ALT: Alanine transaminase level
AST: Aspartate transaminase level
BMI: Body mass index
CI: Confidence intervals
CK: Creatine kinase
ePROs: Electronic patient-reported outcomes
EULAR: European League Against Rheumatism
HAQ: Health Assessment Questionnaire
HCP: Healthcare professionals
ICC: Intraclass correlation coefficients
IIM: Idiopathic inflammatory myopathies
IMACS: International Myositis Assessment and Clinical Studies Group
IMUs: Inertial measurement units
IPAQ: International Physical Activity Questionnaire
IQR: Interquartile range
MMT8: Manual muscle testing, subset of eight muscle groups
MRI: Magnetic resonance imaging
NRS: Numeric rating scale
PHACO: Physical Activity-related Health Competence
PRO: Patient-reported outcomes
REDCap: Research electronic data capture
ROM: Range of motion
SDM: Shared decision-making
SD: Standard deviation
SRM: Standardized response mean
SF-36: Short Form Health Survey
STS: 60 s Sit-to-stand test
T0: Baseline visit
T1: Three months follow-up visit
TSK: Tampa Scale of Kinesophobia
VAS: Visual analogue scale
