# Supplementary material for: Toward digitally supported self-assessment of patients with idiopathic inflammatory myopathies
Source: Arthritis Res Ther. 2025 Feb 22;27:38. doi: 10.1186/s13075-025-03504-z (PMC11846393; doi:10.1186/s13075-025-03504-z)
Supplement: Supplementary file 1 — Supplementary Material 1 [file 13075_2025_3504_MOESM1_ESM.docx]

Supplementary Material


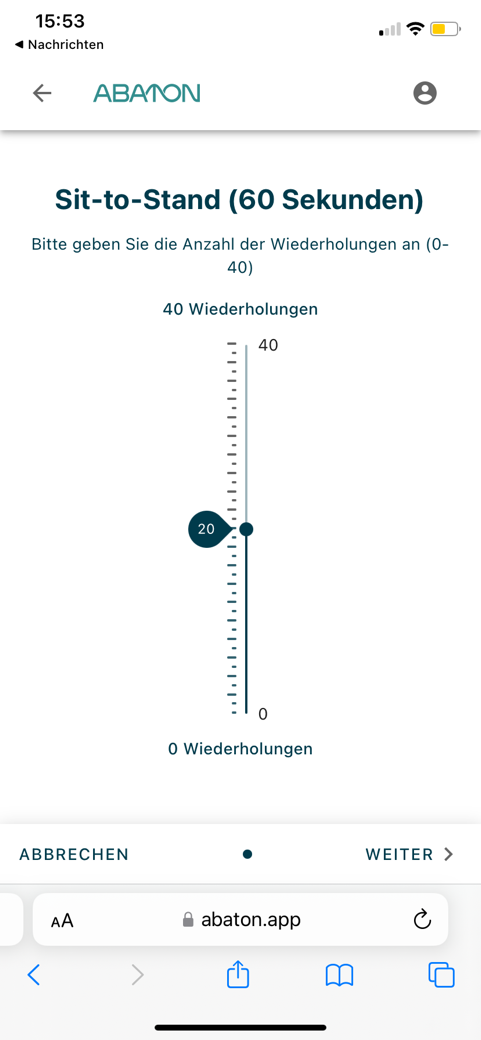


Fig. S1. Screen interface of the medical app ABATON, e.g. patient-reported outcomes (left screen) and exercise data (right screen), adjustable with a slider (0-10 NRS for global disease activity; 0-40 repetitions for the 60-seconds-Sit-to-Stand test).

*Abbreviations: NRS, Numeric Rating Scale; ePROs, electronic patient-reported outcomes.


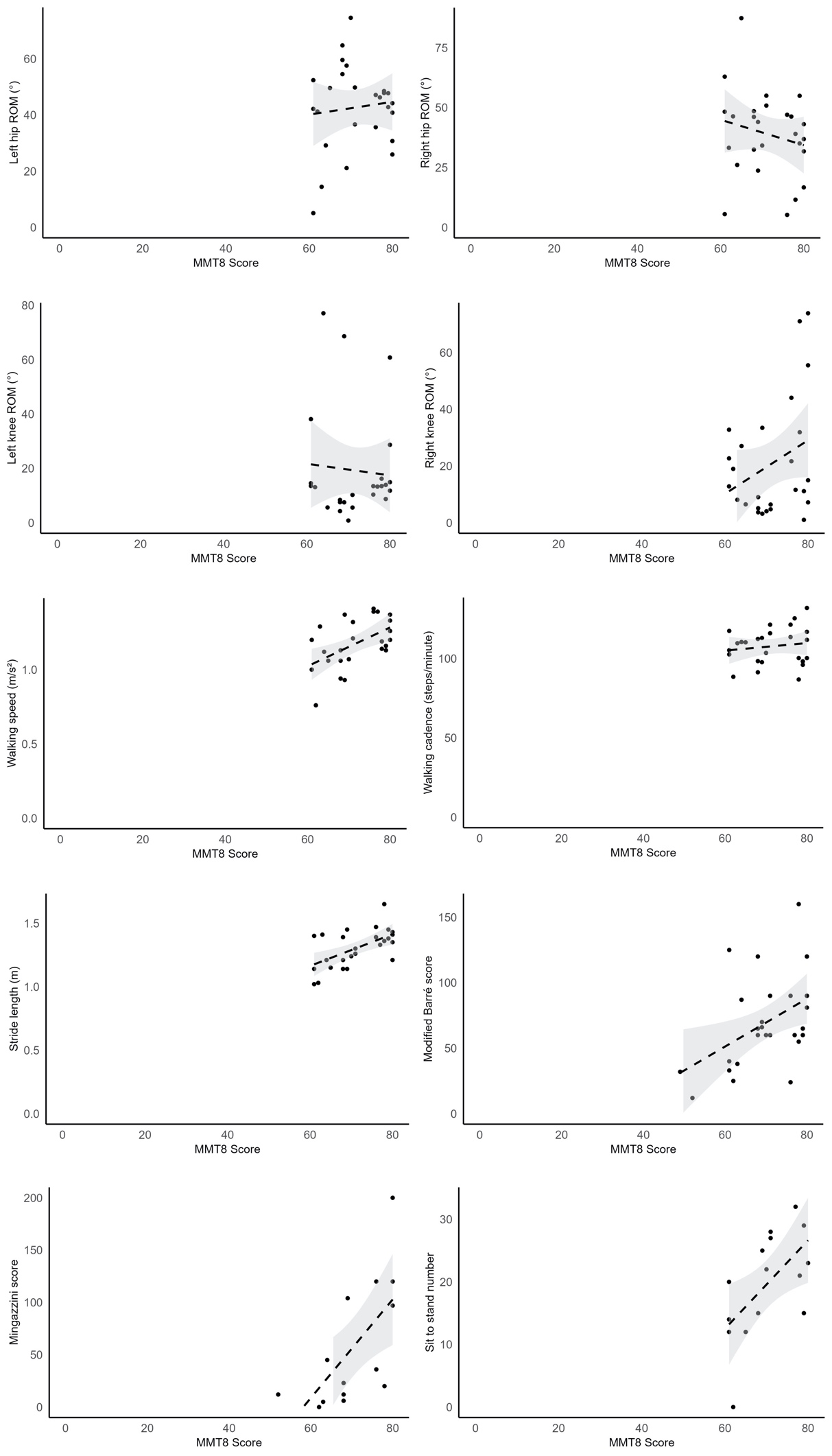


Fig. S2. Graphical representations of the individual correlations of gait parameters in relation to the MMT8 score.

*Abbreviations: MMT8, manual muscle testing; ROM, range of motion.
